# Deficits of Attention and Working Memory in Patients with Gliomas of Supplementary Motor Area

**DOI:** 10.3390/jcm14041229

**Published:** 2025-02-13

**Authors:** Aleksandra Bala, Agnieszka Olejnik, Antonina Gottman-Narożna, Weronika Rejner, Kacper Koczyk, Tomasz Dziedzic, Przemysław Kunert

**Affiliations:** 1 Faculty of Psychology, University of Warsaw, Stawki 5/7, 00-183 Warsaw, Poland; an.olejnik@uw.edu.pl (A.O.); w.rejner@student.uw.edu.pl (W.R.); 2 Department of Neurosurgery, Medical University of Warsaw, Banacha 1a, 02-097 Warsaw, Poland; 3 Doctoral School, Medical University of Warsaw, Żwirki i Wigury 61, 02-091 Warsaw, Poland

**Keywords:** brain tumor, glioma, attention, working memory, neuropsychological assessment

## Abstract

**Objectives:** The effects of brain tumors located in the supplementary motor area (SMA) have so far been described mainly in the context of motor and speech disorders. There are few studies that have considered other cognitive domains, so this study aimed to fill this gap by focusing on examining attention and working memory in a population of patients with gliomas in the SMA region. **Methods:** This study included 50 patients diagnosed with gliomas located in the SMA who have not yet had any treatment and 57 demographically matched healthy individuals. A set of neuropsychological tests was conducted to assess attention and working memory: Digit Span from WAIS-R, Visual Elevator from TEA, Verbal Fluency Test (switching condition), and Color Trails Test (CTT). **Results:** The analyses showed that patients scored lower in most of the evaluated tests and indicators, namely in Digit Span-forward (t = −2.05; *p* = 0.022), Digit Span-backward (t = −2.63; *p* = 0.005), CTT-2 (t = 4.24; *p* = 0.001), CTT-interference (t = 2.31; *p* = 0.012), Visual Elevator-time (t = 1.83; *p* = 0.035), Visual Elevator-accuracy (t = −2.42, *p* = 0.010), and Verbal Fluency-switching (t = −3.41; *p* = 0.001). A significant relationship was also demonstrated between the grade of tumor malignancy and the results achieved in some of the neuropsychological tests. The lateralization of the tumor, the size of the lesion, and the presence of epilepsy did not prove to be particularly significant. **Conclusions:** Due to the significant decline in cognitive performance in terms of attention and working memory, we believe that every patient with a tumor in the SMA should undergo a detailed neuropsychological examination, which will profile their functioning and help tailor the best possible psychological care.

## 1. Introduction

The supplementary motor area (SMA) complex is a region located rostral to the primary motor cortex, which can be divided into two sectors: the proper SMA and the anteriorly located pre-SMA [1]. These areas are richly interconnected with other brain regions, influencing the diverse symptomatology in case of damage, which is called SMA syndrome [2]. Studies on SMA revealed the main connections of this structure with the limbic system, basal ganglia, cerebellum, thalamus, contralateral SMA, superior parietal lobule, and portions of the frontal lobes by inter alia (1) U-fibers connecting the precentral gyrus and the SMA; (2) U-fibers connecting the SMA with the cingulate gyrus and limbic system; (3) the frontal aslant tract connecting the SMA with the pars opercularis; (4) medial fibers connecting the SMA with the striatum; (5) claustrocortical fibers connecting the SMA with claustrum; and (6) SMA callosal fibers connecting this structures in both hemispheres [3,4].

Based on current research and clinical observations, the most is known about the role of the SMA in the context of movement and verbal functions [5,6]. Observations of patients with lesions have clearly shown that damage to this structure may cause deficits in the selection, preparation, initiation, and execution of action, and, in the case of the dominant hemisphere, may cause speech disorders, mainly in the form of deficits in the initiation, fluency, and complexity of constructed utterances [7,8,9]. However, little is known about the relationship between the SMA and other cognitive functions.

Attention and working memory are core cognitive functions that enable individuals to maintain and manipulate information over short periods, crucial for complex decision-making, learning, and everyday functioning [10]. While research on attention and working memory has mostly focused on the prefrontal and parietal regions [11,12,13,14,15,16], the medial frontal cortex—particularly the supplementary motor area complex—has only recently gained recognition for its role in supporting these functions.

The functional connectivity of the SMA with the prefrontal area, specifically the dorsolateral prefrontal cortex (DLPFC) and anterior cingulate cortex (ACC) is one of the neuroanatomical foundations that may explain the involvement of this structure in attention and operational memory processes [17,18].

Tumors located in the SMA region may affect the functions of this structure [2,19]. Due to the scarcity of clinical data that go beyond the classically understood SMA syndrome, it was decided to explore this issue in more detail.

The aim of this study was to focus on the assessment of attention and working memory in a group of patients diagnosed with a tumor in the SMA region. It was hypothesized that a) patients with SMA tumors will obtain lower results in neuropsychological tests than healthy individuals; b) patients with a tumor in the right hemisphere will have a different profile of deficits than subjects with tumors in the left hemisphere; and c) patients with co-occurring epilepsy, higher tumor volume, and higher grade of tumor malignancy will present lower cognitive performance.

## 2. Materials and Methods

### 2.1. Participants

Initial qualification for participation in this study was based on an MRI image indicating the location of the tumor in the supplementary motor area. We preoperatively included and examined sixty-three patients with such description of the neuroimage. The final verification took place after a surgery, based on histopathological examination, which revealed that thirteen patients had other types of lesions: six had meningiomas and seven had metastatic tumors; thus, their data were removed from further analyses. Finally, this study involved 50 patients with glioma tumors located in the supplementary motor area complex and 57 healthy volunteers. Exclusion criteria in both clinical and control groups were as follows: history of any other neurological or psychiatric treatment, drug or alcohol addiction, or any serious disabilities that would impair patients’ ability to complete the neuropsychological assessment properly (e.g., major visual or hearing deficits). Demographic and clinical characteristics of the participants are presented in Table 1. Statistical analyses showed that the groups did not differ in terms of any demographical variable.

### 2.2. Procedure

Each participant was examined individually during a single session before the surgery. The duration of sessions varied between participants, depending on their neurological and physical condition, and lasted between 60 and 90 min. The whole procedure took place in a quiet room in the hospital during the morning hours.

At the beginning of the session, participants were asked to provide demographical information about themselves. Clinical data including details about the type and location of the tumor were obtained from MRI performed during hospitalization and histopathological diagnosis obtained a few days after the procedure.

In order to comprehensively assess cognitive functioning, a neuropsychological examination was performed, during which memory, attention, language functions, visuospatial functions, and executive functions were examined. Additionally, the emotional state of the subjects was also assessed to exclude the potential impact of depression on the obtained results.

All procedures were carried out in accordance with the Declaration of Helsinki and approved by the ethics committee of the Faculty of Psychology at the University of Warsaw (protocol code 15 and date of approval: 5 November 2024). All participants gave informed consent to participate in this study.

### 2.3. Neuropsychological Examination

Digit Span: A subtest from the WAIS-R battery that is used to assess immediate and working memory. The examiner reads a series of numbers, and the subject has to repeat these numbers in the same order (Digit Span-forward) or backward (Digit Span-backwards). The series of digits starts out short but becomes longer over time [20].

Visual Elevator: A subtest from the Test of Everyday Attention battery. It is used to test the ability to maintain and switch attention. It consists of a series of pictures presenting elevator doors marking subsequent floors and arrows indicating the direction of travel. The subject’s task is to count on which floor the elevator will finally stop [21].

Verbal Fluency Test (switching condition): The task was derived from CCAS scale [22] and examines attention and working memory. The test requires the examinee to recall as many words as possible that fit a given category (e.g., animals). In the “switching” condition, the participants are asked to alternately name words from two categories: vegetables and professions. The examinee has one minute to recall as many words as possible that fit the given categories.

Color Trails Test (CTT): A neuropsychological assessment used to evaluate attention, processing speed, and working memory. The test consists of two parts, in each of which the subject must connect circles with consecutive numbers, scattered all over the page. In the first part called CTT-1, the subject must find and connect 25 consecutive numbers. In CTT-2, the circles must be selected taking into account not only the number (ascending) but also the color (alternately once yellow and once pink). The test must be performed as quickly as possible, avoiding errors [23].

### 2.4. Statistical Analysis

Statistical analyses were performed using the Statistical Product and Service Solutions (SPSS) version 29.02 software (IBM Corp., Armonk, NY, USA) for Windows. The Shapiro–Wilk test showed that the data followed a normal distribution and that it was possible to use the parametric tests, as the data also met other requirements. The comparisons between the means of data obtained from patients and healthy volunteers were conducted using Student’s *t*-test for independent samples, and chi2 test was used to compare frequency of distributions of categorical variables. Pearson’s r coefficient was used in the correlation analysis of continuous variables. The η coefficient was used to assess the correlation between nominal and quantitative variables. Results were considered significant when *p*  < 0.05.

## 3. Results

The scores obtained by patients and healthy individuals were compared. After statistical analysis, it turned out that patients with SMA tumors obtained lower results in most of the studied variables. The only variable that was not significantly different was the time of performing the first part of CTT. The effect size was medium for Digit Span (forward and backward), CTT-interference, and Visual Elevator (time and accuracy) and was large for CTT-2 and Verbal Fluency-switching (Table 2).

Next, a univariate analysis of variance (ANOVA) was conducted to examine the effect of oncological malignancy of the gliomas (WHO group 2, 3, or 4) on neuropsychological performance. Significant results were found in three variables: Digit Span-backward, CTT-1, and Visual Elevator-accuracy. In the Digit Span-backward test, the results revealed a statistically significant effect of the group F(2,47) = 4.70, *p* = 0.016, η_p_^2^ = 0.217. Post hoc Scheffe’s analysis showed that participants with gliomas in grade 2 scored significantly higher than those in grade 3, *p* < 0.05. In the CTT-1 test, the results revealed a statistically significant effect of the group F(2,47) = 6.12, *p* = 0.006, η_p_^2^ = 0.290. Post hoc Scheffe’s analysis showed that participants with gliomas in grade 2 scored significantly higher than those in grade 4, *p* < 0.01. In the Visual Elevator-accuracy test, the results revealed a statistically significant effect of the group F(2,47) = 4.44, *p* = 0.021, η_p_^2^ = 0.241. Post hoc Scheffe’s analysis showed that participants with gliomas in grade 2 scored significantly higher than those in grade 3, *p* < 0.05. This indicates that approximately 21.7% of the variance in Digit Span-backward scores, 29% of the variance in CTT-1 scores, and 24.1% in Visual Elevator-accuracy scores can be explained by the tumor type.

The effect of tumor lateralization on scores was also analyzed, and it was found that the only significant difference was present in Visual Elevator-accuracy scores, where subjects with a tumor in the left hemisphere scored higher than those with a tumor in the right hemisphere (t = 1.80; *p* = 0.41). In addition, the results at the level of statistical trend indicated potential differences in Verbal Fluency-switching (L > R; *p* = 0.07), CTT-interference (L < R; *p* = 0.06), and Visual Elevator-time (L < R; 0.07). The tumor volume did not correlate with any measure of cognitive performance (all *p* > 0.05). It is also worth mentioning that taking into account the handedness of patients did not change the results of the neuropsychological tests.

The presence of epilepsy was associated with differences in CTT-1, where patients with a history of seizures took significantly longer to perform the test than those without a history of epilepsy (t = −1.90; *p* = 0.03). Moreover, differences at the level of statistical trend occurred in the CTT-2 (epi < no epi; *p* = 0.06). The performance of other tests was not significantly different.

## 4. Discussion

The SMA complex is involved in many neural processes, including cognitive functioning due to its abundant connections with various brain structures, i.e., the limbic system, basal ganglia, cerebellum, thalamus, contralateral SMA, superior parietal lobe, and prefrontal regions [3]. This study examined how tumors in the SMA complex affected the outcomes of neuropsychological tests that assessed attention and working memory. Because of the limited studies on this topic in the literature so far, it seemed appropriate to address this issue and investigate whether and how the presence of a glioma located in the SMA complex would affect the functioning of patients compared to a demographically matched healthy population.

We found no intergroup differences in the first part of the Color Trails Test (CTT-1), in which participants must quickly find and connect consecutive numbers on a sheet of paper [23]. This test is relatively easy, and, except for the psychomotor speed, it assesses only simple spatial searching and short-time attention focus. In a previous study [24], the functioning of patients with SMA lesions was assessed with the use of tests measuring attention and psychomotor speed, and it also showed no significant differences from the healthy population. In the second part of the Color Trails Test (CTT-2), individuals with a brain tumor performed significantly worse. In addition to simple searching, the test necessitates switching attention between two sequences and engaging working memory to facilitate search by remembering the stages of both sequences [23]. The Verbal Fluency test in the “switching” condition also necessitates switching between two sequences [22]. Correctly performing this task requires efficient attention but also the ability to maintain the alternation of sequences and already spoken words in working memory to avoid repetitions while simultaneously searching semantic memory to update the next words fitting the category. The task is highly engaging and requires the efficient functioning of a neural loop that includes the SMA, as evidenced by other research [24,25,26].

Another test that detected the presence of intergroup differences was the Visual Elevator (both indexes: accuracy and time), in which the subject must calculate on which floor the elevator will stop, taking into account the several changes in the direction of the ride. Correct performance in this test requires the ability to maintain focused attention and efficient working memory [21], and patients with SMA lesions had lower scores in this cognitive domain.

The last task in which patients performed more poorly than controls was the Digit Span test. Patients scored lower on both parts—the one requiring repetition of digits read to them forward and the second, where they repeated numbers backward. The first part requires primarily attentional focus, while the second part additionally requires the engagement of working memory [20].

In summary, this study showed that patients scored lower than healthy controls on all complex and attention-engaging tasks, and achieved similar results only on a relatively simple spatial attention task. It indicates the important role of the SMA in higher-level attentional functioning.

Information on the relationship between working memory and the SMA is very limited. There are studies that demonstrated the engagement of this area based on neuroimaging [27,28]; direct brain stimulation during awake surgery [29], which was performed only in two individuals; case series [30]; and intergroup comparisons of patients with lesions in the SMA and healthy controls [24], with the limitation that the group of patients was very heterogenous and included only twelve subjects, which makes the conclusions not sufficiently representative of the broader population.

Nevertheless, considering these data, we can assume that both the SMA and pre-SMA are involved in working memory, although most likely in distinct ways. Taking into account neural connections, the SMA probably contributes mostly to motor working memory, particularly in the context of remembering and executing complex motor sequences. With its connections to the prefrontal cortex and limbic structures [3,4], the pre-SMA is likely involved in higher-order working memory processes, cognitive aspects of task sequencing, and complex attention, such as voluntary target selection, task maintenance, and attention switching between items [31,32].

There is even less information available about the SMA’s involvement in attentional tasks, with only some experimental data [33] and case reports [34] available, but, to our best knowledge, no cohort studies have yet been conducted. The pre-SMA’s connections with the prefrontal cortex [3,4] may support attentional control and the dynamic updating of working memory, while the connection with the ACC facilitates error monitoring [35,36] and adjustments in attentional focus. In contrast, the SMA’s connections with primary motor and premotor areas underscore its role in tasks involving sustained attention to motor sequences and working memory involving motor responses [31].

Due to the nature of glioma growth [37] and the specificity of the clinical group described in this study, it was not possible to isolate subjects with lesions exclusively within the proper SMA and pre-SMA and compare their performance, but this is an important and interesting direction for further research.

Subsequent analyses examined the impact of particular clinical variables on neuropsychological performance. It turned out that the degree of tumor malignancy was significant for three tasks: Digit Span-backward, CTT-1, and Visual Elevator-accuracy. In each of these tasks, patients with grade 2 tumors scored more favorably, which is in line with other studies showing that patients with brain tumors with higher grades exhibited cognitive impairments more often compared to patients with lower grades [38,39]. This may be because tumors with higher grades of malignancy are characterized by a higher rate and aggressiveness of tumor growth than low-grade gliomas, which in turn makes neuronal reorganization and compensation of the resulting damage more difficult; they may also have a greater irritant effect on the surrounding tissue [40,41,42].

Interestingly, only one significant difference was detected between patients with tumors in the left and right hemispheres. The explanation may be that attention and working memory are functions performed by both hemispheres [43] but also that the left and right SMA are functionally interconnected [4]; thus, damage to any one of them damages the entire network in which they are involved and the lateralization of the damage may be of secondary importance. There are several studies that have detected an association between the side of the tumor and the profile of cognitive disorders [44,45,46], but these were papers focusing on tumors in locations other than the SMA.

There are some studies that have shown a link between tumor size and the level of cognitive functioning [47,48,49,50]. In the present study, the tumor volume did not correlate with any measure of cognitive performance. The result is not intuitive, as it would seem that a larger tumor mass would more strongly damage the supplementary motor area and thus more strongly damage patients’ functioning. This effect can perhaps be explained by the fact that the SMA is so important in the neural network involving attention and working memory [24] that it makes little difference whether this area is damaged by a large tumor or a small one because damage to it affects the functioning of the entire network.

Contrary to the hypotheses, the presence of epileptic seizures did not prove to significantly worsen patients’ functioning; statistical differences occurred in only one of the analyzed indicators. The reason for this is probably that most of the patients had seizures for a relatively short time, as these were subjects for whom an epileptic seizure was the reason for reporting to the doctor and learning that they had a brain tumor, so their exposure to the damaging effects of seizures was short. As numerous studies have shown, taking antiepileptic drugs [51] and a long duration of epilepsy [52] can negatively affect patients’ cognitive functioning. Thus, the short history of seizures in the studied group may have been too insignificant for differences to be noticeable.

## 5. Conclusions

This study provides valuable insights into the cognitive effects of SMA damage, emphasizing its role in attention and working memory. The findings underscore the importance of assessing these functions in patients with SMA gliomas to better understand their cognitive deficits. From a practical perspective, this study highlights the necessity of early neuropsychological assessment for patients with SMA gliomas. Tailored preoperative cognitive stimulation programs focusing on enhancing attention and working memory could improve patients’ performance and perhaps shorten the postoperative time for cognitive recovery. Future research should include advanced neuroimaging techniques, such as resting-state fMRI or DTI, and the results of direct electrical stimulation under awake conditions to further elucidate the neural mechanisms underlying these cognitive deficits. These approaches could also aid in distinguishing the specific contributions of the SMA and pre-SMA to working memory and attentional control, expanding the description of the classically understood SMA syndrome and paving the way for more precise therapeutic interventions.

## Figures and Tables

**Table 1:** Demographical and clinical data.

	Clinical Group	Control Group	*p*-Value
Sex: W/M	27/23	33/24	>0.05
Age M (SD)	42.66 (12.31)	41.92 (13.65)	>0.05
Years of education M (SD)	14.02 (2.78)	14.78 (2.80)	>0.05
Handedness: R/L	44/6	50/7	>0.05
Tumor grade: G2/G3/G4	23/15/12	N/A	N/A
Hemisphere: L/R	29/21	N/A	N/A
Seizures: yes/no	29/21	N/A	N/A
Tumor volume in cm^3^ M (SD)	30.76 (32.70)	N/A	N/A

**Table 2:** Comparison of neuropsychological tests.

	Clinical Group M (SD)	Control Group M (SD)	Student’s t Value	*p*-Value	Cohen’s d
Digit Span—forward	5.77 (2.23)	6.84 (2.22)	t = −2.05	**0.022**	−0.47
Digit Span—backward	5.05 (2.13)	6.44 (2.43)	t = −2.63	**0.005**	−0.60
CTT-1	43.10 (19.98)	46.39 (25.57)	t = −0.59	0.278	−0.14
CTT-2	105.93 (50.28)	65.43 (22.86)	t = 4.24	**0.001**	1.05
CTT-interference	1.45 (0.71)	1.10 (0.53)	t = 2.31	**0.012**	0.55
Visual Elevator—time	5.06 (2.24)	4.37 (1.27)	t = 1.83	**0.035**	0.41
Visual Elevator—accuracy	8.03 (2.16)	9.07 (1.34)	t = −2.42	**0.010**	−0.61
Verbal Fluency—switching	12.71 (5.22)	17.00 (2.28)	t = −3.41	**0.001**	−1.04

*Legend*: significant scores are in bold.

## Data Availability

The data presented in this study are available on reasonable request from the corresponding author.
